# Dimensional model of adolescent personality pathology, reflective functioning, and emotional maltreatment

**DOI:** 10.3389/fpsyt.2023.1302137

**Published:** 2024-01-10

**Authors:** Karin Ensink, Mélissa Deschênes, Michaël Bégin, Laurie Cimon, Thomas Gosselin, Marissa Wais, Lina Normandin, Peter Fonagy

**Affiliations:** ^1^École de Psychologie, Université Laval, Québec, QC, Canada; ^2^Département de Psychologie, Université de Sherbrooke, Sherbrooke, QC, Canada; ^3^Département de Médecine Sociale et Préventive, Université Laval, Québec, QC, Canada; ^4^Research Department of Clinical, Educational and Health Psychology, University College London, London, United Kingdom

**Keywords:** mentalizing, personality organization, emotional abuse, childhood maltreatment, adolescence

## Abstract

**Introduction:**

Childhood emotional abuse (CEA) is a recognized risk factor for adolescent mentalizing challenges. However, there's limited understanding about how CEA might influence personality development and elevate the risk of adolescent personality pathology. A deeper grasp of these pathways is crucial, given that adolescence is a pivotal developmental phase for identity integration, personality consolidation, and the emergence of personality disorders. As the emphasis shifts to dimensional perspectives on adolescent personality pathology, the spotlight is increasingly on adolescents' evolving personality organization (PO). Within this framework, personality disorder manifestations stem from inherent vulnerabilities in PO. A comprehensive understanding of how CEA leads to these inherent vulnerabilities in PO can inform enhanced interventions for at-risk adolescents. Nonetheless, our comprehension lacks insight into potential pathways to PO, especially those involving external factors like maltreatment and individual traits like mentalizing. This study sought to bridge these gaps by employing latent factor analysis and structural equation modeling to explore connections between emotional maltreatment, adolescent mentalizing, and PO.

**Methods:**

A community-based cohort of 193 adolescents (aged 12–17) took part in self-report assessments: the Childhood Experience of Care and Abuse Questionnaire (CECA.Q), the Reflective Functioning Questionnaire for Youth (RFQ-Y), and the Inventory for Personality Organization for Adolescents (IPO-A).

**Results:**

The structural equation model revealed a significant direct influence of CEA on both RFQ-Confusion and PO, and a noteworthy direct effect of RFQ-Confusion on PO. Remarkably, the model accounted for 76.9% of the PO variance. CEA exhibited a significant indirect impact on PO through RFQ-Confusion, which was accountable for 52.3% of the CEA effect on PO, signifying a partial mediation by mentalizing.

**Discussion:**

These insights carry substantial clinical implications, especially for devising integrated, trauma-informed strategies for adolescents with personality pathologies. This is particularly relevant for enhancing mentalizing and bolstering personality consolidation among adolescent CEA survivors.

## 1 Introduction

Adolescence is a pivotal period for identity integration and personality consolidation (1–3), coinciding with the emergence of personality disorders (4–8). In line with the evolving shift toward a dimensional model of adolescent personality disorders (9), the concept of personality organization (PO) serves as a valuable lens for pinpointing dispositional vulnerabilities tied to the heightened risk of developing adolescent personality disorders. A profound understanding of these vulnerabilities in PO could enhance clinical interventions and diminish the risk of these disorders. Childhood maltreatment, particularly CEA, is a documented risk factor for the onset of personality disorders during adolescence. CEA, also termed psychological abuse, encompasses caregiver behaviors such as derogatory name-calling, insulting, threats, and the deprivation of love, support, and guidance, all of which can detrimentally impact a child's mental wellbeing. Such maltreatment impedes the growth of socio-cognitive abilities like mentalizing (10–14) and also hampers the evolution of self-capacities, resulting in self-related issues, including challenges in maintaining a stable sense of self and identity (15, 16). Preliminary findings suggest a mediating role of mentalizing between CEA and identity diffusion in adolescents and indicate an association between mentalizing difficulties and identity diffusion in adults diagnosed with personality disorders (17, 18). Even though identity remains a core component of PO, the broader repercussions of CEA on PO are yet to be comprehensively understood. Significant knowledge voids persist, especially concerning potential pathways through which CEA and intrinsic traits like mentalizing influence PO. To the best of our awareness, no prior studies have examined the interrelations among CEA, mentalizing, and PO in the adolescent population. Consequently, our study endeavors to bridge these knowledge lacunae, elucidating the pathways from CEA through mentalizing to PO.

### 1.1 Childhood maltreatment, emotional abuse, and its clinical fallout in adolescence

Adolescence represents a particularly vulnerable phase for survivors of childhood maltreatment due to the myriad of challenges and susceptibilities that coincide with the transition into adulthood. During this period, those who have experienced childhood maltreatment are at heightened risk for numerous adverse outcomes, including delinquency, becoming a victim or perpetrator of violence (19–22), engaging in high-risk sexual behaviors leading to pregnancy (19–23), substance abuse (23–25), academic failure (26), and a range of psychological issues (27, 28). A deeper exploration is required to understand the maltreatment-related dispositional vulnerabilities in PO that elevate the risk of these negative trajectories during adolescence.

Recent research indicates that CEA can have particularly detrimental effects on the development of self-capacities and personality, further correlating with a heightened probability of personality pathology in both adolescents and adults. Notably, CEA has been linked to features of borderline personality in adolescents (29–32) and in adults (33, 34), especially when CEA interplays with inherent temperamental vulnerabilities (29, 31). Strikingly, individuals diagnosed with borderline personality disorder (BPD) are almost 14 times more likely to disclose experiences of childhood maltreatment in comparison to non-clinical counterparts, with CEA and neglect being the predominant forms reported (35). A staggering 84% of those with BPD recall instances of neglect and CEA before reaching the age of 18 (35). Another concerning observation is that adolescents who engage in Non-Suicidal Self-Injury (NSSI) consistently report higher levels of maltreatment compared to their healthy peers (36). Among various forms of maltreatment, CEA and neglect appear more closely tied to self-harm than physical or sexual abuse. Amplifying this concern, a meta-analysis conducted by (37) established a significant association between childhood maltreatment and elevated impulsivity levels, with CEA demonstrating the most profound effect size.

Preliminary research suggests that PO mediates the relationship between CEA and symptoms of borderline personality disorder (9). Yet, significant knowledge gaps persist concerning the impact of CEA on PO in adolescents, as well as the potential role of mentalizing.

### 1.2 Personality organization

Building on enduring theories around the development of PO and personality disorders (38), there has been a notable shift from categorical perspectives on personality pathology toward dimensional models that emphasize self and interpersonal capacities. This dimensional approach to personality disorders has been incorporated into the Alternative Model for Personality Disorders in the fifth edition of the Diagnostic and Statistical Manual of Mental Disorders [DSM-5; (4)] and the 11th edition of the International Classification of Diseases [ICD-11; (39)]. Within this framework, personality disorders are characterized by structural impairments in self and personality functioning (40). Moreover, symptoms of personality disorders, such as self-harm, are perceived not merely as isolated behaviors, but as manifestations stemming from underlying personality vulnerabilities (9). There is a growing consensus that these dimensional models offer a more developmentally attuned and clinically pertinent perspective (2, 41, 42).

Rooted in psychodynamic object relations theory (43, 44), PO adopts a dimensional lens, focusing on intrinsic psychological “structures” believed to underpin both typical personality functioning and personality pathology. At the heart of these structures are “internal object relations,” postulated to emerge in childhood from the amalgamation of internalized representations of early interactions with attachment figures and the associated emotions triggered by such relations. As these patterns become increasingly ingrained over time, they lay the groundwork for a higher-order psychological structure termed “identity,” reflecting an individual's comprehension of self and significant others. Complementing this structure are other facets of personality organization, encompassing moral values, regulation of aggression, and reality testing. Disturbances in PO have shown associations with personality disorders in both adults [e.g., (45)] and adolescents (1, 46). On the healthier end of the PO spectrum, individuals exhibit cohesive identities, robust reality testing capabilities, well-developed moral systems, and minimal aggression. Conversely, those on the severe end are characterized by identity diffusion, compromised reality testing, weak moral foundations, and heightened aggression.

### 1.3 Mentalizing

Mentalizing is a multi-faceted socio-cognitive skill central to our understanding of ourselves and others, as well as our interpersonal interactions. By enhancing self-awareness and rendering the reactions of oneself and others comprehensible and foreseeable (47), mentalizing strengthens social bonds and attachment connections. Additionally, mentalizing aids in the physiological regulation of distress (11) and is linked to reduced cardiovascular response during attachment-related stress scenarios.

Developmentally, the foundation of mentalizing lies in early attachment relationships wherein children are recognized and treated as beings with minds. Within these relationships, children cultivate the ability to articulate their emotions and thoughts, and to interpret the actions of others through the lens of underlying mental intentions and states (48). In line with this perspective, the mentalizing skills of children and adolescents are often influenced by the observable mentalizing capacities of their parents, both in direct interactions and in discussions with and about the child (12, 13).

However, experiences of maltreatment can significantly hinder the development of mentalizing and its constituent elements across all age groups—children, adolescents, and adults (10, 12–14, 49). Such impairment might arise because the trauma of maltreatment intensifies the individual's sense of isolation, creating a feeling that their inner thoughts and feelings are uniquely theirs and unshared (50). Among various forms of maltreatment, CEA stands out for its especially detrimental impact on adolescents' mentalizing capacities (30, 32, 51–53). Instances of CEA where parents misinterpret and misattribute their children's reactions and intentions, and subsequently belittle, harm, bewilder, and manipulate them, prove particularly deleterious for the normal development of mentalizing. Such experiences generate uncertainty in the child about their own emotional and mental states, as well as the intentions of those around them. Impaired mentalizing creates a broad-based susceptibility to various mental health challenges in adolescents (53) and plays a significant role in the interpersonal issues and emotional volatility typically associated with personality disorders (54).

To the best of our understanding, only one prior study has explored both mentalizing difficulties and PO (55), discovering associations between both and disconnected, highly insensitive maternal behaviors. An amalgamation of challenges in mentalizing and PO was correlated with aggressive intrusive and withdrawn behaviors. These findings align with our proposition that an integrative framework encompassing difficulties in both mentalizing and PO is needed to understand individual vulnerabilities predisposing to personality disorders.

The present study's objective was to employ latent construct analysis to discern CEA and PO constructs and utilize structural equation modeling to elucidate potential pathways bridging CEA, mentalizing, and PO. Drawing upon prior studies (18, 30, 32, 53), we postulated that CEA would correlate with heightened challenges in mentalizing, typified by confusion regarding mental states. Further, based on initial observations (9), we anticipated a link between CEA and increased PO challenges. Given the dearth of preceding research focusing on mentalizing and PO, we refrained from asserting a definitive hypothesis. However, relying on past findings that pinpoint mentalizing difficulties as being associated with pronounced identity diffusion (17, 18), we cautiously hypothesized a connection between mentalizing challenges and increased problems of PO Moreover, though no earlier studies have examined pathways involving CEA, mentalizing, and PO, insights from prior findings (18)—which suggest mentalizing as a mediator between CEA and identity diffusion—led us to tentatively propose that mentalizing would act as a partial mediator in the relationship between CEA and PO. Recognizing the multi-faceted nature of PO, we did not anticipate complete mediation by mentalizing, postulating that CEA might influence PO dimensions through channels distinct from mentalizing.

## 2 Methodology

### 2.1 Participants and procedure

The study included a sample of 193 adolescents ranging in age from 12 to 17 years, with a mean age of 14.89 years (SD = 1.47). The gender distribution revealed a majority of girls (68.50%; *n* = 132) and a minority of boys (31.50%; *n* = 61); none of the participants identified as intersex. In terms of ethnicity, the majority of participants were White (92.80%). Smaller proportions identified as Black (2.44%), Asian (1.46%), Hispanic (0.70%), Native American (0.52%), and other ethnicities (2.28%). Recruitment took place at local high schools. Eligibility for the study was based on age, requiring participants to be between 12 and 17 years. Those with intellectual disabilities were excluded from participation.

### 2.2 Measures

#### 2.2.1 Emotional maltreatment

Childhood maltreatment was assessed using the Childhood Experience of Care and Abuse Questionnaire (CECA.Q) (56). This self-report measure evaluates various dimensions of childhood maltreatment perpetrated by caregivers, encompassing neglect, antipathy, psychological abuse, role reversal, physical abuse, and sexual abuse. A latent factor for emotional maltreatment was derived from the neglect (16 items; α = 0.87), psychological abuse (34 items; α = 0.88), and antipathy (16 items; α = 0.91) subscales. Items are scored on a five-point Likert scale, ranging from 0 (never) to 4 (often), for each caregiver, with higher scores indicating a more severe maltreatment history. The CECA.Q exhibits robust psychometric properties, including convergent and construct validity and test-retest reliability (>0.70). Its sound psychometric properties have been confirmed in both clinical (57) and community samples (56).

#### 2.2.2 Personality organization

The Inventory for Personality Organization in Adolescents (IPO-A) (46) is an adolescent-adapted self-report tool crafted to assess PO following the structural model of personality (44). The questionnaire gauges five dimensions of PO: stability of self and other perceptions (11 items; α = 0.84), instability of objectives (5 items; α = 0.75), aggressiveness (11 items; α = 0.87), reality testing (6 items; α = 0.88), and moral values (9 items; α = 0.79). Items are scored on a 5-point Likert scale from 1 (never true) to 5 (always true), where higher scores indicate greater personality pathology. The French adaptation of the IPO-A has been validated in a community cohort of adolescents and young adults (46).

#### 2.2.3 Reflective functioning

The Reflective Functioning Questionnaire for Youth (RFQ-Y) (58, 59) is a concise self-report tool aimed at quantifying adolescents' reflective functioning or mentalizing capabilities. It encompasses three scales: Confusion and uncertainty about mental states (11 items; α = 0.88), Curiosity regarding mental states (8 items; α = 0.72), and Certainty about others' mental states (6 items; α = 0.81). Items are rated on a 6-point Likert scale from 1 (strongly disagree) to 6 (strongly agree). Higher scores on the individual scales, respectively, suggest increased uncertainty, interest, or certainty about the respondent's and others' mental states. The validity and psychometric attributes of the French version were established in a community sample of adolescents (59).

### 2.3 Data analysis

Preliminary bivariate correlations were used to examine the associations between childhood maltreatment, reflective functioning and personality organization. The correlations were then used to identify relevant variables for the subsequent analysis. In order to test the mediational role of confusion about mental states (RFQ-C) in the association between emotional maltreatment and personality pathology, structural equation modeling (SEM) was used. The measurement model was twofold. First, a latent variable (emotional maltreatment) predicting psychological abuse, neglect was computed. Residuals were allowed to correlate in the final model. Then, a second latent variable predicting personality organization variables (aggression, identity diffusion, reality testing and moral functioning) was computed. The correlations between the residuals were also allowed. The structural model aimed at examining the direct effect of emotional maltreatment on personality organization as well as the indirect effect through confusion about mental states (RFQ-C). Because some variables were non-normally distributed, we used a robust estimator (MLR). Different fit indices were used to test the adequacy of the model: the Comparative Fit Index (CFI), the Tucker-Lewis Index (TLI), the root mean square error of approximation (RMSEA) and the standardized root mean square residuals (SRMR), and the ratio of chi-square to degrees of freedom. Guidelines suggest that values above 0.95 for the CFI and TLI (60) and values below 0.05 for the RMSEA and SRMR, as well as a ratio of chi-square to degrees of freedom (χ^2^/*df* ) of <3, indicate an excellent fit (61, 62). Missing data was handled using the built-in full information maximum likelihood method in Mplus 8.10. Using a conservative effect size estimation based on Cohen's recommendations (63), a sample of 201 participants was necessary to detect significant effects with a statistical power of 0.80 (64). However, with the actual effect size being higher than the estimation, the number of participants needed to achieve 0.80 was significantly lower.

## 3 Results

### 3.1 Preliminary analyses

Bivariate Pearson correlations assessed the relationships among childhood maltreatment, PO, and reflective functioning (refer to Table 1). Our findings revealed moderate to strong correlations among various IPO-A dimensions. However, Instability of goals stood out, as it did not show any significant correlation with other dimensions, leading to its exclusion from the latent PO factor. Similarly, the CECA-Q subscales pertaining to CEA, specifically Negligence, Antipathy, and Psychological abuse, exhibited moderate to strong interconnections. Notably, among the RFQ-Y factors, Confusion about mental states displayed the most robust and consistent links with the study's variables. As a result, the Confusion factor was selected to explore the mediational role of RF in the SEM. Additionally, the study noted only minimal or negligible correlations between physical and sexual abuse with other studied variables. Consequently, these maltreatment types were set aside in subsequent analyses.

**Table 1:** Bivariate Pearson correlations between variables of study.

**Variables**	**1**	**2**	**3**	**4**	**5**	**6**	**7**	**8**	**9**	**10**	**11**	**12**	**13**
**IPO-A subscales**													
1. Stability in sense of self and others													
2. Aggressivity	0.41^***^												
3. Moral functioning	0.69 ^***^	0.45^***^											
4. Reality testing	0.46^***^	0.33^***^	0.44^***^										
5. Instability of goals	−0.07	0.10	−0.05	0.01									
**CECA-Q subscales**													
6. Antipathy	0.29^***^	0.28^***^	0.35^***^	0.29^***^	0.02								
7. Negligence	0.08	0.20^***^	0.20^**^	0.17^*^	0.10	0.75^***^							
8. Psychological abuse	−0.01	0.14	0.05	0.08	0.07	0.35^***^	0.34^***^						
9. Role reversal	0.22^**^	0.06	0.25^***^	0.31^***^	0.04	0.32^***^	0.26^***^	0.25^***^					
10. physical abuse	0.13	0.16^*^	0.09	0.17^*^	−0.01	0.23^**^	0.18^*^	0.07	0.18^*^				
11. Sexual abuse	0.10	0.10	0.15^*^	0.12	−0.00	0.08	0.09	0.05	0.05	0.09			
**RFQ-Y subscales**													
12. Confusion	0.65^***^	0.51^***^	0.64^***^	0.49^***^	0.12	0.33^***^	0.21^***^	0.13	0.32^***^	0.17^*^	0.19^**^		
13. Interest/curiosity	0.25^***^	−0.21^**^	0.12	0.06	−0.26^***^	−0.01	−0.21^**^	−0.24^***^	0.03	0.05	0.06	−0.03	
14. Certainty	0.36^***^	0.21^**^	0.39^***^	0.22^**^	−0.27^***^	0.22^**^	0.12	−0.05	0.21^**^	0.10	0.01	0.19^**^	0.42^***^

N, 193. IPO-A, Inventory for Personality Organization in Adolescents; CECA-Q, Childhood Experience of Care Questionnaire; RFQ-Y, Reflective Functioning Questionnaire for Youth. ^*^p < 0.05; ^**^p < 0.01; ^***^p < 0.001.

### 3.2 Structural equation modeling

**Figure 1:**
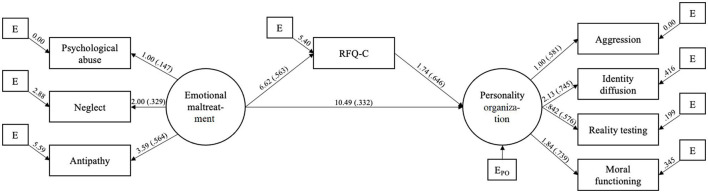
SEM with direct effect of emotional maltreatment on personality organization and indirect effect through RFQ-confusion. ( ) Standardized coefficients (ß); all associations are significant at *p* < 0.05; correlations between residuals not displayed.

The full results of the SEM are available in Figure 1. According to the above-mentioned guidelines, the model showed a good- to excellent fit with a ratio of χ^2^/*df* > 3 (21.84/3), a RMSEA of 0.086, a SRMR of 0.035, a CFI of 0.978 and a TLI of 0.931. First, the measurement model for emotional maltreatment indicated that the latent variable explained 2.2% of the variance of psychological abuse, 10.8% of neglect and 31.8% of antipathy. The measurement model regarding personality organization showed that the latent variable explained 33.8% of the variance of aggression, 55.6% identity diffusion, 33.2% of reality testing and 54.6% of moral functioning. Then, the structural model indicated a significant direct effect of emotional abuse on RFQ-Confusion (ß = 0.570, *p* = 0.021). In addition, the structural model indicated a significant direct effect of emotional maltreatment on personality organization (ß = 0.343, *p* = 0.001) as well as a significant direct effect of RFQ-Confusion on personality organization (ß = 0.645, *p* = 0.001). Furthermore, emotional abuse showed a significant indirect effect on personality organization through RFQ-Confusion (*b* = 11.679, *p* < 0.001). The indirect effect was responsible for 52.3% of the total effect of emotional abuse on personality organization and was consistent with a partial mediation. 76.9% of the variance of personality organization was explained by the model.

## 4 Discussion

The primary objective of this study was to elucidate the interrelations between CEA, mentalizing, and PO among adolescents through latent factor analysis and SEM. Our structural equation model revealed a pronounced direct influence of CEA on both RFQ-Confusion and PO. Moreover, a significant direct impact of RFQ-Confusion on PO was observed. Highlighting the role of partial mediation by mentalizing, CEA demonstrated a consequential indirect effect on PO via RFQ-Confusion, accounting for 52.3% of CEA's total influence on PO. Impressively, our model captured 76.9% of PO's variance. Overall, the results underscore that the interplay between CEA and mentalizing challenges can elucidate a significant portion of the variance in PO. As posited, CEA's association with augmented disturbances in PO is partially attributed to its detrimental effect on mentalizing, which in turn was associated with heightened challenges in PO.

This study's primary contribution lies in identifying the clinical repercussions of CEA on both mentalizing and PO. This encompasses adverse effects on identity diffusion, moral functioning, aggression, and reality testing. These findings resonate with preliminary data (9), which linked CEA with augmented challenges in PO and highlighted the mediating role of PO between CEA and BPD traits. Employing the PO model afforded a more comprehensive understanding of the repercussions of CEA on personality maturation, thus broadening the scope beyond earlier research that primarily examined CEA's negative influence concerning identity diffusion (17, 18).

The insights garnered hold profound clinical ramifications, illustrating how CEA predisposes adolescents to susceptibilities in mentalizing and PO, which manifest as increased identity diffusion, potential moral development challenges, elevated aggression, and enhanced reality testing difficulties. Understanding the deleterious ramifications of CEA on PO can pave the way for designing interventions that more effectively mitigate these vulnerabilities during this pivotal developmental phase because it is also at this developmental stage that the capacity to construct an identity based on a self-narrative emerges (65, 66). It is only after the emergence of metacognitive capacities sufficient to create an integrated, evolving, coherent story of the self that an individual becomes able to represent themselves coherently to others and to themselves, drawing together their significant life experiences (67). Narrative identity is a level of personality that is more idiographic, dynamic, and contextual than traits and characteristic adaptations and is influenced by how parents talk with their children (68). By creating a story and a set of meanings around personal attributes, we create meaning around events in our lives and interconnect past, present, and expected experiences that collectively generate a unit of experience around William James' “Me” as a separate individual a person refers to when talking about their personal experiences that feels sustainable over time (69). The expected coherence needed to sustain wellbeing is provided by these self narratives (67). Across adolescence, a depth in autobiographical reasoning grows, which allows individuals to begin to construct a life story or narrative identity (68). It is the development of social cognition, more particularly mentalizing capacities, that should come on stream at this time, that may cause in those with experiences of CEA gaps in their ability to bind together experiences and dispositional traits into a coherent story around the self begins to show (70) and impact on PO become observable. Narrative coherence increases with age and those with features attributed to BPD manifest lower levels of narrative coherence as well as identity diffusion (71, 72). Notably, of the individual symptoms contributing to a BPD diagnosis, identity disturbance is most strongly associated with the total number of suicide attempts in adolescence (73). From a therapeutic standpoint, this accentuates the need for trauma-aware interventions to encompass a focus on PO to counteract these susceptibilities and the aftermath of CEA.

CEA's association with heightened confusion concerning mental states, and the subsequent deficits linked to increased PO disturbances, underscores a concerning pathway. Simplified, adolescents affected by CEA experience challenges in PO primarily because CEA impedes the natural evolution of mentalizing which in turn we assume is critical to creating a coherent self-narrative. This hindered mentalizing capacity probably also further elevates preexisting difficulties with PO. This extends prior findings (18), which pinpointed mentalizing as a mediator between CEA and identity diffusion within a clinical adolescent cohort. Our research expands on this by demonstrating that, amidst CEA, uncertainties about mental states not only accentuate identity diffusion but also adversely impact other PO dimensions, such as moral development, reality testing, and aggression regulation probably through limiting the capacity for coherent self-narratives. This leaves these adolescents prone to personality-intrinsic vulnerabilities. In situations marked by CEA, this muddled sense of mental states could further destabilize reality testing, inducing confusion about discerning the real from the unreal. This aligns with and broadens the scope of existing studies (30–32), highlighting the role of RF in personality challenges among adolescents with CEA histories. For instance, the association between CEA and pathological personality traits was delineated by RF (30–32). Our study's contribution lies in its detailed exploration of specific traits, like mentalizing difficulties and PO, that magnify the vulnerabilities tied to CEA in adolescents.

Additionally, alongside its established detrimental effect on identity diffusion, CEA was found to influence moral development, reality testing, and aggression. While existing literature hasn't directly explored CEA's relationship with moral development, research has identified that women with trauma-induced PTSD from childhood display reduced altruistic moral reasoning, heightened self-concern, and diminished empathic role-taking (74). A longitudinal study also noted that while CEA and childhood neglect corresponded with reduced empathy, childhood physical abuse and exposure to domestic violence resulted in amplified empathy (75). Given that CEA often targets a child's psychological self, it may particularly obstruct the natural evolution of prosocial responses, such as moral development and empathy, which are inherently geared toward fostering cooperation (76). Concerning CEA's ties with aggression, the results echo previous findings indicating that psychological abuse correlates with escalated relational aggression during adolescence, and that childhood neglect is a potent predictor of violent behaviors in adults (77, 78). Although scant literature connects childhood maltreatment with reality testing, one notable study identified that maltreatment severity during childhood related to increased reality testing issues, encompassing reality distortion and perception uncertainties, in at-risk adolescent groups (79). Given that CEA often involves negating a child's lived experience and might even involve intentionally muddling their recollections, it's plausible that this would compromise their grip on reality. Further, this concept of reality testing challenges parallels the “Pretend Mode” in Mentalization Based Therapy (54)—a pre-mentalizing state that conflates reality with fantasy, neglecting real-world engagements or forming reality-aligned plans.

The findings from this study underscore the critical importance of trauma-informed interventions that address vulnerabilities associated with CEA and aim to mitigate the risk of personality disorders in adolescents. Central to these interventions should be strategies that bolster the development of mentalizing capacities related to both self and others. This scaffolding can be instrumental in alleviating the confusion CEA-exposed adolescents often experience, thereby facilitating the process of personality consolidation. Moreover, addressing trauma-induced instabilities in the representations of self and others, as well as integrating trauma-related aggression through heightened self-awareness, can be critical in fostering personality consolidation in these adolescents. Furthermore, fostering epistemic trust can potentially remedy the moral challenges associated with CEA that often hinder successful interpersonal integration. This approach, in turn, can be beneficial for personality consolidation and in reducing the risk of personality disorders. Lastly, addressing trauma-induced challenges in discerning reality and understanding tendencies to escape into fantasy or “pretend modes” can be vital. Addressing these tendencies, which might impede adaptive functioning and engagement with age-specific developmental tasks, may further promote personality integration and reduce vulnerabilities predisposing to personality disorders.

### 4.1 Study strengths and limitations

One of the significant strengths of this study lies in its application of multivariate statistics within the Structural Equation Modeling (SEM), enhancing the study's internal validity. Another strength is the relatively large sample size. However, there are also notable limitations. The study's cross-sectional and correlational design restricts the generalizability of its findings. Additionally, while self-report measures offer a window into adolescents' perceptions and experiences, they may not be as objective as observer-rated metrics or experimental tasks. Factors such as self-awareness and the desire for social acceptability might influence participants' responses (80). Moreover, given that this research was grounded in a community sample, generalizing the findings necessitates caution. Another limitation is the underrepresentation of adolescent boys in the study sample, indicating a potential gender bias. Future research should aim for greater gender inclusivity, ensuring more comprehensive insights and broader generalizability.

### 4.2 Conclusion

This study enriches our understanding of the vulnerabilities tied to CEA, particularly concerning mentalizing and PO in adolescents. The proposed pathway model underscores that CEA not only has a direct impact on adolescent PO impairments but also exerts an indirect influence by triggering mentalizing challenges, notably confusion. This, in turn, escalates PO deficiencies. The observed patterns align with the notion that the link between CEA and PO impairments is partially mediated by mentalizing difficulties. From a trauma-informed therapeutic standpoint, initiatives that bolster mentalizing abilities and address the trauma effects on PO can be instrumental in curtailing the risk of personality disorders among adolescents exposed to CEA.

## Data availability statement

The raw data supporting the conclusions of this article will be made available by the authors, without undue reservation.

## Ethics statement

The studies involving humans were approved by Les Comités d'éthique de la recherche avec des êtres humains de l'Université Laval (CÉRUL). The studies were conducted in accordance with the local legislation and institutional requirements. Written informed consent for participation in this study was provided by the participants' legal guardians/next of kin.

## Author contributions

KE: Conceptualization, Supervision, Writing – original draft, Writing – review & editing. MD: Writing – original draft, Writing – review & editing. MB: Formal analysis, Writing – original draft. LC: Writing – original draft. TG: Writing – original draft. MW: Supervision, Writing – review & editing. LN: Writing – review & editing. PF: Writing – review & editing.
